# Supplementary material for: Overwintering Camelina and Canola/Rapeseed Show Promise for Improving Integrated Weed Management Approaches in the Upper Midwestern U.S
Source: Plants (Basel). 2023 Mar 15;12(6):1329. doi: 10.3390/plants12061329 (PMC10056582; doi:10.3390/plants12061329)

Supplemental Figure S1: Neighbor-joining tree for 58 overwintered winter canola/rapeseed accessions

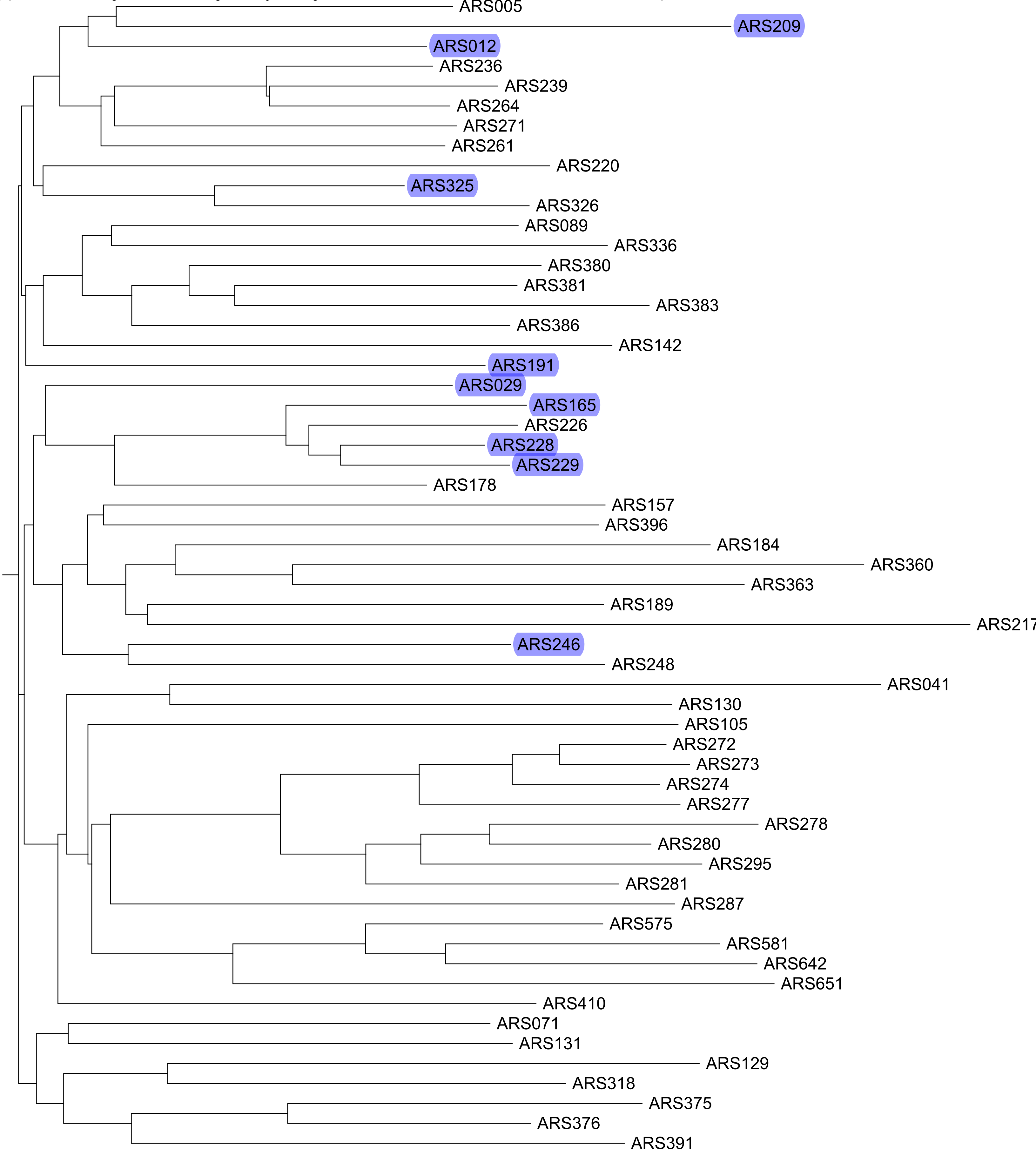

Supplement: Supplementary file 1 [file plants-12-01329-s001.zip › Supplemental Figure S1.pdf]
